# Influence of Fibre Fill Pattern and Stacking Sequence on Open-Hole Tensile Behaviour in Additive Manufactured Fibre-Reinforced Composites

**DOI:** 10.3390/ma16062411

**Published:** 2023-03-17

**Authors:** Alessia Teresa Silvestri, Ilaria Papa, Antonino Squillace

**Affiliations:** Department of Chemical, Materials and Industrial Production Engineering, University of Naples Federico II, 80125 Naples, Italy

**Keywords:** 3D printing, additive manufacturing, CFF, continuous filament fabrication, composites, onyx, continuous fibre, open hole, mechanical characterisation

## Abstract

Additive manufacturing has revolutionised the field of manufacturing, allowing for the production of complex geometries with high precision and accuracy. One of the most promising applications of additive manufacturing is in the production of composites, which are materials made by combining two or more substances with different properties to achieve specific functional characteristics. In recent years, the use of Continuous Filament Fabrication (CFF) in additive manufacturing has become increasingly popular due to its ability to produce high-quality composite parts which have fibres with a complex orientation and high curvature. This paper aims to investigate the influence of fill pattern and stacking sequence on the open-hole tensile strength of composites manufactured using CFF and made of an innovative matrix composed of nylon and short carbon fibres, i.e., Onyx, and with continuous carbon fibre as reinforcement. By systematically varying the fill pattern and stacking sequence, we aim to identify the optimal combination that can achieve the highest open-hole tensile strength in these composites. The results of this study will provide valuable insights into the design and manufacture of high-strength composites using additive manufacturing. Open-hole strength and elastic properties are strongly influenced by the infill strategy and stacking sequences adopted, and show different failure modes. The results also point out a technological issue characterising the process and indicate some guidelines for designing and manufacturing 3D printing composites.

## 1. Introduction

The increasing interest in innovative and high-performing materials has made composites a frequent subject in various fields of research due to their excellent strength/weight ratio and the multiplicity of areas in which they can be applied (aerospace, automotive, sports, construction). Many studies describe the traditional fabrication technologies used to make these materials, from protrusion to filament winding, since composites are obtained through different phases such as polymer matrix and fibre reinforcements [1,2]. On the other hand, other studies concern the optimisation of the processes for the existing techniques in order to improve performance and, at the same time, to obtain lightweight materials. However, these conventional techniques require moulds which increase costs, and these techniques are limited to the performances reached up to now. Additive Manufacturing (AM) is a promising evolution in this field, allowing the fabrication of composite materials in a polymeric matrix both economically and flexibly [3,4]. It works by processing layer by layer with the aid of a computer and a 3D design file. An additive manufacturing process can produce highly flexible, functional and even complex structures. It is often used to create geometrically-multifaceted designed prototypes and components. It is attracting recognition as it reduces ride time for product development, as well as reducing the demand for costly instruments. Among the existing techniques, FDM or fused deposition modelling, is the most widely-used 3D printing technique as it is fast and low-cost, and allows complex-shaped parts to be obtained easily and quickly. As with other AM technologies, FDM adds material layer by layer through an additive process starting directly from a computer-aided design model, saving energy, raw material costs and waste [5,6,7]. Moreover, fabrication time can be reduced significantly, especially when complex geometry is required. FDM Composite technology has the benefit of changing the fibre orientation in complex curvatures, as in the case of the area around the boundary of a gap such as a hole or a junction, to moderate the consequences of stress concentration. It appears that FDM technology, which allows the manufacturing of customised laminates, is an up-and-coming technique which can be integrated successfully with other technologies to provide the best solution for manufacturing 3D-printed tailored systems [8,9].

To create efficient components and increase the market share of FDM parts, it is essential to generate elements with balanced qualities which meet specific constraints. The FDM procedure has numerous factors that have a significant effect on built features. All these factors influence the connection between the deposited layers [10,11]. A component having the required characteristics can be made by choosing a proper combination of process factors. Part quality needs to be improved to compete with traditional manufacturing processes and to advance the benefits of the additive manufacturing process [12]. Therefore, considerable importance will be given to the process parameters such as the thickness of the layer, the orientation of the construction, the direction and width of the raster, the air gap and the filling density, which are the most frequently analysed process parameters [13].

Despite their advantages over the conventional manufacturing process, FDM parts often have lower mechanical properties. First of all, the materials used for printing are mainly thermoplastic polymers, which are weaker than metals. Additionally, the printed parts’ anisotropic material properties and the layer-by-layer print deposition process produce voids due to poor adhesion among the layers, resulting in lower tensile strengths. Therefore, it is necessary to improve the processes in order to improve the mechanical properties of the final product. Melenka et al. studied the influence of the volume of fibre reinforcement in samples printed using the fibre fill strategy and demonstrated that an increase in the volume of fibre reinforcement results in an increase in the stiffness and ultimate strength of the test samples [14]. Caminero et al. studied the improvement in impact strength of 3D composites by changing building orientation, layer thickness, fibre type and volume content [15]. Other researchers have investigated the mechanical behaviour of these kinds of printed parts in order to improve their performance [16]; however the parts inevitably need to be connected or assembled by opening holes. Therefore, among the various mechanical tests carried out, it is also crucial to evaluate the stress concentrations around discontinuity that can produce early failures such as pin and rivet holes [17]. The introduction of such holes results in stress concentration and a decrease in the net section. Nonetheless, it is customary in aerospace engineering to establish notched design allowable strengths based on gross section stress to address diverse stress concentrations (such as fastener holes, free edges, flaws, and damage, among others) that are not explicitly modelled in the stress analysis. Open-hole tensile strength is a key mechanical property that is critical in determining the structural performance of composite materials.

Open-hole tensile testing (OHT) in isotropic and traditional composite materials has been analysed to a significant extent [18]. Of course, open holes in anisotropic materials can produce a more complex break and damage mechanism, greatly reducing strength [19,20,21,22]. It was shown that the mechanical features of an open-hole composite are constrained by stacking sequences, hole dimension, layer thicknesses, specimen size and fibre volume [23]. Unlike conventional composites, 3D components do not involve a successive machining process to create holes or discontinuities. This means the damage post-process is minimal in comparison to that obtained by using traditional machining processes such as matrix breaking and delamination. However, the use of 3D printing technology in composite manufacturing has presented new challenges that need to be addressed. Sansei et al. studied the influence of hole diameter on the OHT strength. By varying the geometry suggested by the D5766 standard [24,25], Shafighfard et al. investigated the mechanical behaviour of different open-hole geometries, proposing three different design methodologies and studying different sizes and geometries of the hole [26]. Dickson et al. conducted a study on the mechanical behaviour of open-hole composites produced using Continuous Filament Fabrication (CFF) technology and with Nylon as the matrix material. The researchers explored the effects of various factors, such as fibre direction, type, and volume fraction, on the mechanical properties of the composites. Their findings revealed that carbon fibre was the optimal choice for achieving the highest mechanical strength values [27]. Indeed, carbon-fibre-reinforced composites are known for their exceptional strength and stiffness, making them suitable for demanding aerospace applications [28]. 

However, one of the remaining challenges is the optimisation of the deposition pattern and stacking sequence in 3D-printed composites with carbon fibres. The fibre fill pattern and lay-up sequence are critical factors that affect the mechanical behaviour of 3D-printed composites, especially in open-hole tensile strength applications. Given the innovative and unexplored nature of this material and the importance of in-depth knowledge of the mechanical performance of composites, the authors wanted to test the material’s behaviour in order to fill a knowledge gap on the topic. This work therefore aims to present a comprehensive study on the effects of fill pattern and stacking sequence on the open-hole tensile strength of 3D-printed carbon fibre composites, providing valuable insights for the development of high-performance composite structures.

Three different kinds of hole reinforcements made using the concentric strategy and three kinds of stacking sequences made using the isotropic strategy were adopted for the manufacturing of open-hole tensile specimens. The mechanical results and the failure modes can be used to design and manufacture 3D composites with carbon fibres for applications that require high strength and durability.

## 2. Materials and Methods

In the present work, “Onyx” and Carbon Fibres (CF) were used as matrix and reinforcement phases, respectively, to produce test composite specimens (supplied by Markforged CA). The Onyx matrix is a particular nylon blend patented by Markforged, i.e., nylon filled with short carbon fibres, and therefore it is already a kind of composite. The raw material is supplied in 150 cm^3^ spools. Before printing, the spools must be stored in a dry box due to the hygroscopic nature of the nylon. The short CF does not only serve as reinforcement and improve the mechanical performance of the blend, but it also changes the behaviour of the material during the cooling phase by causing less thermal deformation. As a result, the dimensions of the additively manufactured parts correspond as closely as possible to the model produced in CAD. Compared to pure nylon, onyx is approximately 3.5 times more resistant, has a higher hardness and an HDT (Heat Deflection Temperature) of 140 °C [29]. Carbon fibres are supplied in the form of a continuous strand of about 1000 fibres with a diameter of 10 μm. The continuous fibre reinforcement can be printed in long strands, creating composite parts that are many times stronger and stiffer than the bare onyx. The presence of long carbon fibres guarantees additional strength, reliability and durability to the object and allows for a wide variability of applications and, consequently, increasingly widespread use. Furthermore, thermoplastic polymers reinforced with long fibres have an optimal relationship between stiffness and weight, allowing them to withstand the most severe test and to offer a significant reduction in weight at a competitive cost [30].

### 2.1. Experimental Process (3D Printing Machine and Process)

The Markforged X7 3D printer by LabCaMP2 University of Naples with a build volume equal to 300 × 270 × 200 mm was used. It is equipped with two independent extruders. The first nozzle builds up the plastic matrix, and the second one wraps the fibres. The extruders move along the x and y axes, while the print bed moves along the z axis. The process starts with uploading the STL file into the associated software (MarkforgedEiger™). It is possible to set some process parameters such as materials and deposition strategy; other parameters such as printing speed and temperature are set as optimal according to the material. The type and amount of reinforcement determine the final properties of the parts. As with conventional processes, the highest strength and stiffness are achieved with continuously reinforced composites [31]. However, it should be emphasised that perfect alignment of continuous fibres is still difficult, time-consuming and costly to achieve in conventional composite manufacturing processes. The additive CFF process is able to solve and overcome the above-mentioned weak point in the production of continuous fibre composites, given that the alignment, direction and orientation of the continuous fibres can be controlled and arranged using this technology [32].

### 2.2. Experimental Campaign

**Figure 1:**
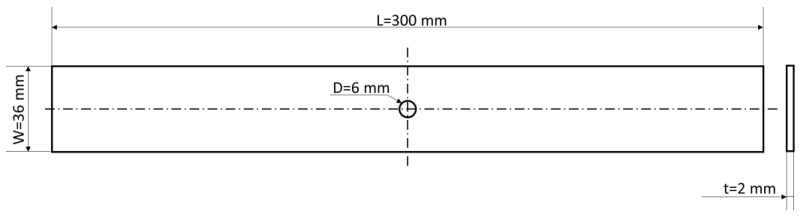
Schematic representation of the Open-Hole specimen according to the ASTM standard D5766, with its main dimensions.

This work aims to investigate the influence of the hole reinforcement and stacking sequence on the Open-Hole tensile strength of 3D-printed composites in Onyx and carbon fibre by means of CFF technology. For this reason, samples were designed in accordance with the ASTM D5766 standard (specifically, configuration A) [25]. The specimen was rectangular flat panels with dimensions of 300 × 36 × 2 mm (Figure 1). A circular hole with a fixed diameter (D) of 6 mm was designed in the CAD file and created directly during the deposition phase. In this way, the drilling phase is avoided, preventing several defects such as delamination, irregular holes and rough edges [33]. Specimens were printed with a fixed “width to diameter ratio” (that is the ratio of the sample width to the hole diameter) and a constant “diameter to thickness ratio”, W/D = 6 and D/t = 3, respectively, as suggested by the standard.

**Figure 2:**
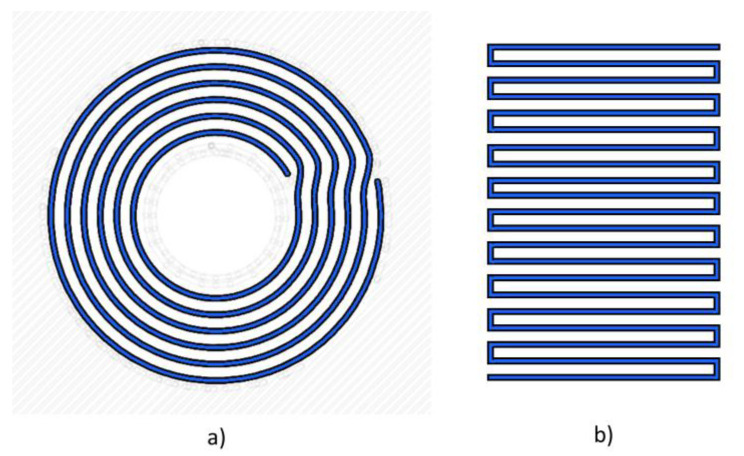
Fibre fill pattern: (**a**) concentric, (**b**) isotropic.

The test specimens are made of 16 layers, the first and the last of which are printed entirely in Onyx. The layer height is 0.125 mm; the “wall layers”, i.e., the contouring layers, are set at 4. As explained in the introduction, by using CFF technology it is possible to design and print composite components which have fibre which is characterised by complex orientation and high curvature, and which can be applied to enhance mechanical properties near geometric discontinuities, such as the hole in the present study. This is made possible by choosing different reinforcement deposition strategies; the ones of interest are illustrated in Figure 2. The reinforcement strategy can also be indicated as a fill pattern and can be isotropic or concentric. In the latter case, it is possible to set the number of walls to reinforce the inner discontinuity, i.e., the number of rings around the hole or the external feature. In the present experimental campaign, the authors aimed to investigate the influence of reinforced hole specimens, and therefore a fibre concentric fill pattern was selected to print the CF rings. This pattern is shown in Figure 2a. On the other hand, the influence of the stacking sequence was also studied using the fibre isotropic fill pattern illustrated in Figure 2b.

The experimental campaign consisted of two macro-typologies of specimens, the first printed using the concentric fibre strategy and the second using the isotropic fibre strategy. Each macro-category consisted of 3 different kinds of samples, with a different number of concentric fibre rings or different stacking sequences, for a total of 6 different kinds of specimens, as summarised in Table 1, where the subscript “s” means “symmetrical”.

**Figure 3:**
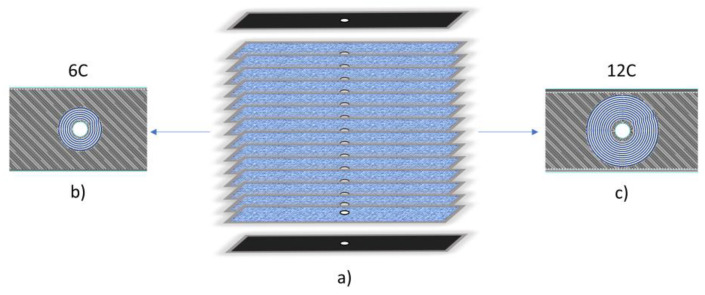
(**a**) Schematic representations of the composite sample composed of 16 layers; scheme of inner layers printed with fibre around the inner hole in concentric fill pattern samples: (**b**) 6 rings of CF, (**c**) 12 rings of CF.

In the concentric pattern category, the samples called “Onyx” consisted entirely of layers of full Onyx matrix. The matrix was always printed alternatively at 45° and −45°. The other two specimen types, shown in Figure 3a,b, were printed with concentric and continuous carbon fibres around the inner centred hole, for a total of 6 and 12 rings around the hole. Therefore, their specimen identification names are “6C” and “12C”. In the figure, the carbon fibres are represented by blue lines, and the Onyx matrix by darker zones. Twelve rings are the maximum number of concentric carbon-fibre rings that can be used for the chosen geometry (36 mm width). The six concentric rings are, therefore, the middle point between the maximum number of concentric fibre rings and the sample without hole reinforcement.

**Figure 4:**
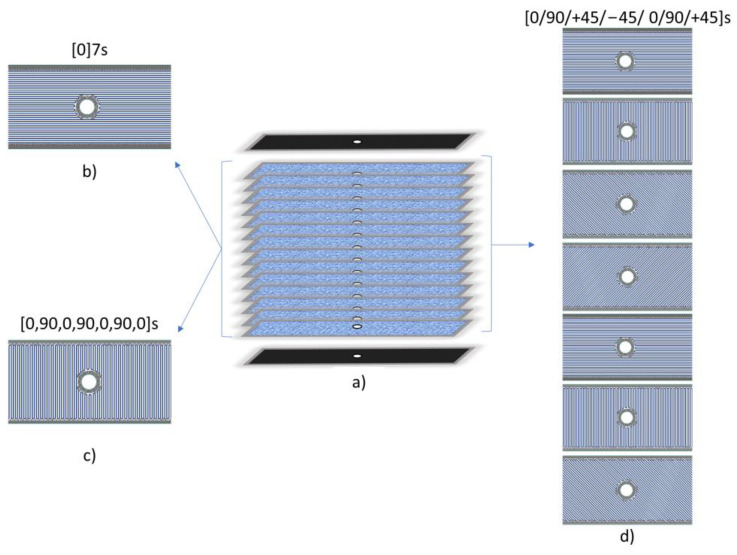
(**a**) Schematic representation of the composite sample composed of 16 layers; representation of the inner layers printed using a fibre stacking sequence in an isotropic fill pattern: (**a**) sample arrangement, (**b**) 0° CF, (**c**) 0/90° CF, (**d**) 0/90/45° CF.

For the remaining three specimens, carbon-fibre-reinforced composites with aligned carbon fibre were investigated. The percentage fibre volume was 70%, which is the practical limit of volume percentage reinforcement, beyond which there is not enough matrix to support the fibres in the composite [31]. Therefore, three different stacking sequences were investigated:

(i) CF were printed in the same direction for each layer, achieving a unidirectional (UD) lamina, i.e., [0°]_7S_, (Figure 4a);
(ii) a composite panel was printed with a [0,90,0,90,0,90,0]_S_ stacking sequence (Figure 4b);
(iii) a composite panel was printed with a [(0/90),(+45,−45),(0/90/45)]_S_ stacking sequence (Figure 4c).

Hereinafter, for the sake of brevity, the specimens are referred to as the “0°”, “0/90°” and “0/90/45°” samples, respectively.

### 2.3. Sample Characterisation: Open-Hole Tensile Test

**Figure 5:**
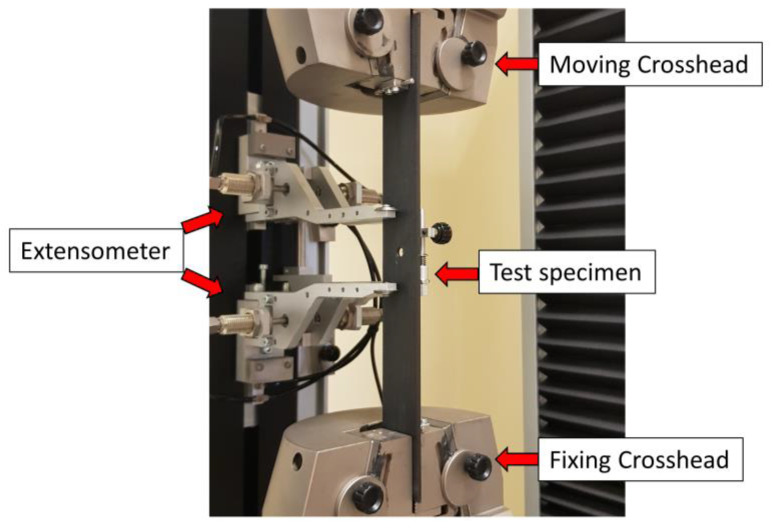
Open-Hole tensile test experimental set-up.

The open-hole tensile tests were carried out at room temperature using a universal testing machine (Galdabini QUASAR 50, Galdabini SPA, Varese, Italy) located in LabcAMP2 in accordance with ASTM D5766. The Quasar 50 is equipped with a 50 kN load cell and a micron extensometer to measure the deformation up to the rupture, and it is a displacement-controlled testing machine. The speed of the crosshead was 3 mm/min. It is equipped with software that allows the tests to be set up correctly and the condition of the tests to be checked at any moment. Five samples were tested for each experimental configuration. Figure 5 shows the experimental set-up.

The ultimate open-hole tensile strength is calculated according to the following formula (Equation (1)):

(1) $$F^{OHT}=\frac{P^{max}}{A}$$

where *F^OHT^* (MPa) is the ultimate tensile strength in the test direction, *P^MAX^* (N) is the maximum force carried by the sample before failure and *A* (mm^2^) is the gross cross-sectional area, disregarding the hole. The elastic properties, i.e., the stiffness, were indicated as K. 

**Figure 6:**
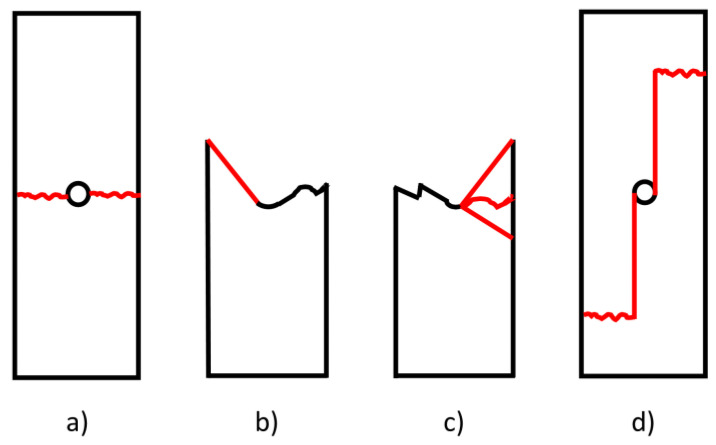
Acceptable Open-hole tensile failure modes: (**a**) Lateral Gage Middle, (**b**) Angled Gage Middle, (**c**) Multi-mode Gage Middle and (**d**) Longitudinal Splitting Gage Middle.

Failures that do not occur at the hole are not acceptable failure modes and the relevant data is marked as invalid. Failure is often strongly influenced by delamination and the failure mode may exhibit a great deal of delamination. The standard proposes a three-letter notation to describe failure modes: the first character should describe the failure type, the second should describe the failure area and the last should describe the failure location [25]. In this test method, the test can be considered valid if the failure takes place in the “Gauge Middle” (code GM). Even although the failure modes detected are normally lateral, angled or multi-mode, in the present work “longitudinal splitting” failure types were also detected. A magnified picture around the failure is used to highlight and show the failure mode. Some of the acceptable failure modes are illustrated in Figure 6. In Figure 6a, the LGM failure indicates that the laminate tensile failure occurs laterally across the centre of the hole. In Figure 6b, the AGM code suggests that the laminate fails in tension at the hole, but the remnants of the angle cross the lateral centreline of the hole. In the case of the MGM code (Figure 6c), the laminate fails at the hole and exhibits multiple modes of failures in various sub-laminates, this kind of failure is characterised by extensive splitting and delamination. The SGM failure (Figure 6d) indicates that the laminate fails at a hole along the longitudinal direction. The mode of failure may be found to vary on different sides of the hole.

## 3. Results and Discussion

**Figure 7:**
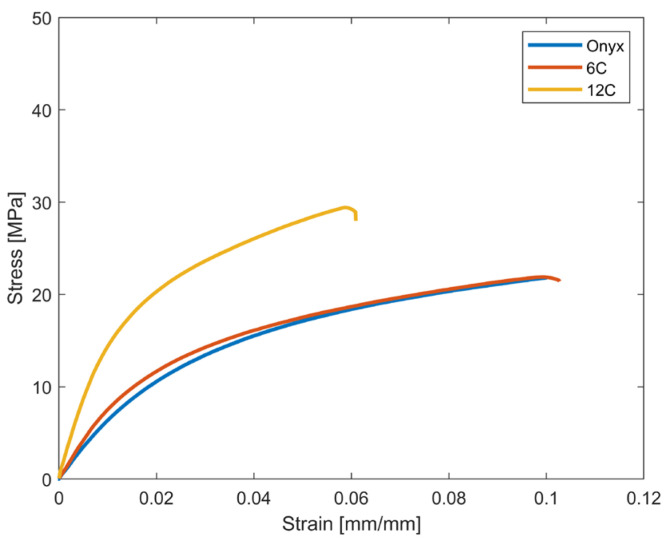
Open-Hole tensile test results for samples printed with concentric infill pattern: stress-strain curves.

**Figure 8:**
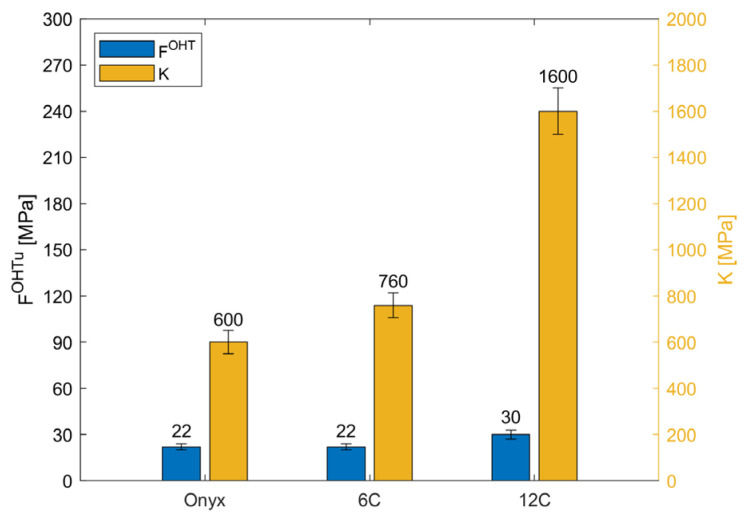
Open-Hole tensile test results for samples printed with concentric infill pattern: mean value of the open-hole strength and stiffness.

Figure 7 shows the stress-strain curves for samples printed using the concentric fibre deposition strategy. For the sake of brevity, one curve representative of each type of tested sample is illustrated. In Figure 8, the mean value of the open-hole strength and its standard deviation are given.

The carbon fibres printed with a concentric pattern around the hole were introduced to reinforce the discontinuity and enhance the open-hole strength. However, the results revealed that concentric rings of CF did not affect the mechanical behaviour of the sample when compared to the sample printed in full Onyx. Specifically, the *F^OHT^* and the elastic properties are very similar in the Onyx and 6c samples, and are close to the tensile strength measured for samples without holes and made in full Onyx [29]. The performances are improved with the introduction of 12 CF reinforcement, with an increase in OH tensile strength of 23% and an approximately 52% increment in stiffness. On the other hand, displacement decreased with the increase in CF concentric reinforcement and stiffness; specifically, as the number of CF rings increased, the displacement decreased from 0.1 to 0.08 to 0.06 mm/mm.

**Figure 9:**
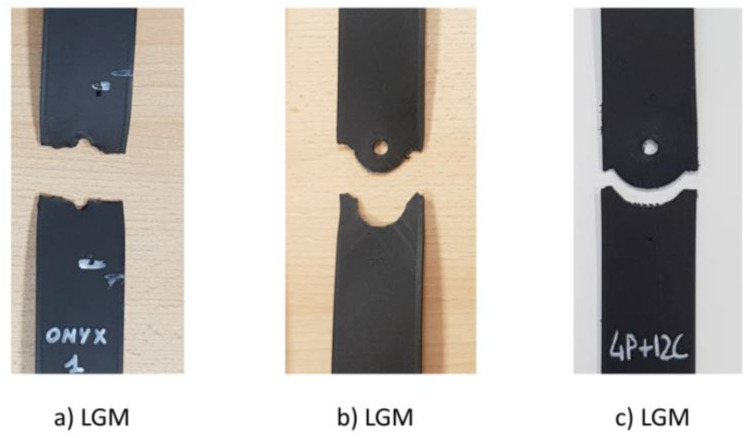
Failure modes in fibre concentric fill pattern: (**a**) LGM in Full Onyx samples, (**b**) LGM in 6C samples and (**c**) LGM in 12C samples.

Concerning the failure mode, the magnified pictures of the tested samples are shown in Figure 9. In this category, one failure mode is observed: LGM, i.e., Lateral Gage Middle. Specimens 6C and 12C have experienced a Lateral failure type at the last CF ring, the sixth and the twelfth, respectively, leading to a matrix-fibre debonding.

Both the mechanical behaviour and the failure modes can be explained considering that printing the samples with the concentric CF pattern around the inner discontinuity leads not only to a reinforcement of the hole, but also to an increment in the size of the hole. Therefore, it is possible to evaluate the specimen printed with concentric CF as the same as that printed with full Onyx but with a larger hole.

**Figure 10:**
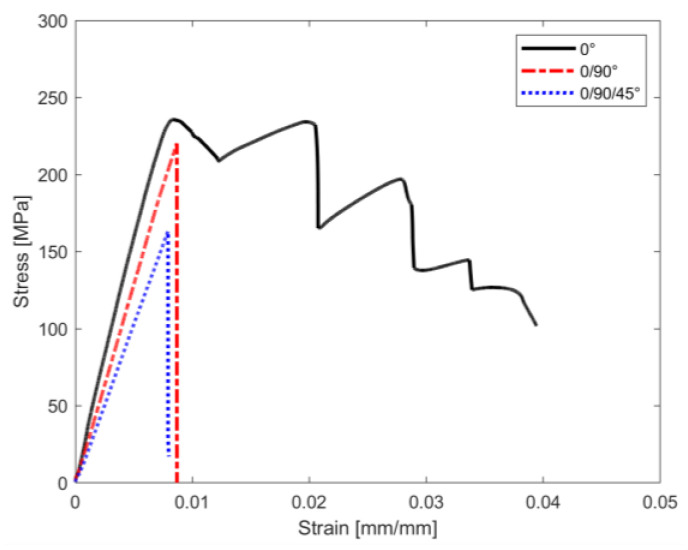
Open-Hole tensile test results for samples printed with isotropic infill pattern: stress-strain curves.

**Figure 11:**
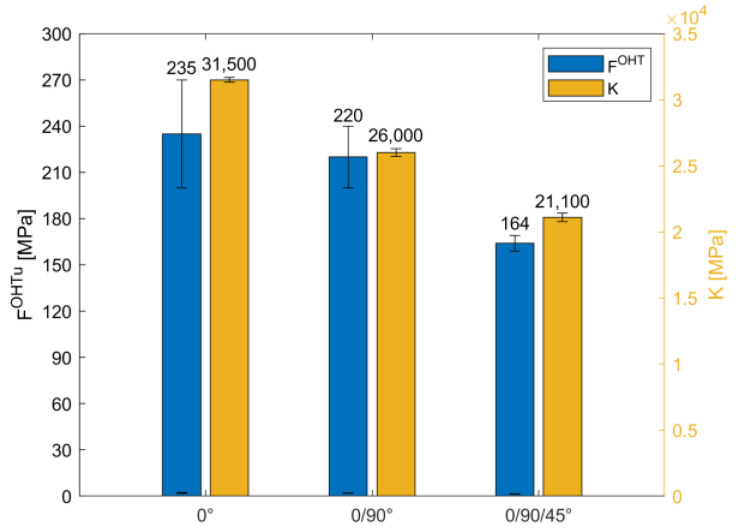
Open-Hole tensile test results for samples printed with isotropic infill pattern: mean value of the open-hole strength and stiffness.

Concerning the second macro-category, i.e., specimens printed using the isotropic deposition strategy, the results of the OH tensile tests are shown in Figure 10 and Figure 11.

The first consideration to be made is that the mechanical responses of samples printed using the isotropic pattern are quite different from the responses of samples printed using the concentric strategy. Printing using an isotropic pattern, and so using continuous and aligned carbon fibres regardless of the fibre orientation, led to a high increment in both open-hole strength and stiffness, at least 633% and over 1500%, respectively, in comparison with the full Onyx samples.

Concerning the influence of the laminate lay-up sequence, as the complexity of the stacking sequence increases, the open-hole strength decreases, achieving the maximum value for UD samples, i.e., 0°, in agreement with the literature in which the maximum tensile strength occurs at the fibres arranged in the direction of load application [34]. Moreover, the 0° specimens experienced progressive failure, so after the initial damage the samples can sustain further loads, exhibiting the highest displacement at failure. Holes are known to cause local stress concentrations that lead to cracking of the matrix, delamination, detachment of the fibre-matrix and micro-buckling of the fibres after the onset of micro-cracking and crack propagation. Therefore, after the initial damage, when the load continues to be applied, the microcracks become more and more and dense until they become visible macrocracks. Macrocracks occur very close to ultimate failure. 

The other two types, i.e., 0/90° and 0/90/45°, experienced catastrophic failure, with the ultimate open-hole tensile strength at the highest displacement. The open-hole ultimate strength in the 0° and 0/90° samples is almost the same, with a *F^OHT^* reduction of only 7% in the 0/90° samples when compared to the 0° samples. Concerning the 0/90/45° specimen, *F^OHT^* reduction reaches 30% in comparison with 0°. Regarding the elastic properties, the major elastic property is the stacking sequence complexity, the minor property is the stiffness.

**Figure 12:**
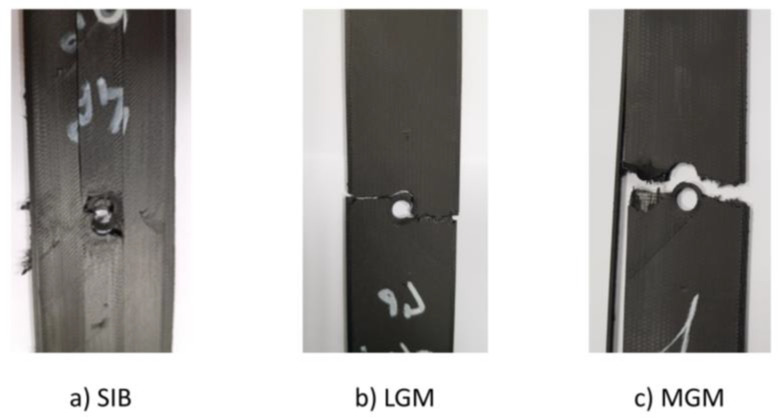
Failure modes in isotropic samples: (**a**) SIB in 0° samples, (**b**) LGM in 0/90 samples, and (**c**) MGM in 0/90/45 samples.

However, some consideration must be given to the failure modes of isotropic samples (Figure 12), bearing in mind that for this test method the only valid failures are those observed in the “Gage Middle” area (indicated as “GM” in the second and third placeholders).

Looking at Figure 12, it can be seen that the failure types are different; in particular the 0° specimens have an invalid failure mode: the fracture extends longitudinally (indicated by the identification code “longitudinal splitting”, i.e., “S”) throughout the specimen even inside the mechanical wedge grips (indicated by the identification code “inside the grip”, i.e., “I”). Even though it appears that the 0° laminate has achieved the best mechanical performance, the failure mode experienced indicates that it is not compliant with the standard (ASTM D5766). This failure is due to the fibre alignment affecting the interfacial adhesion between the reinforcement and the matrix. Printing CF in the same orientation (0° in the present study) in each layer printed with CF can cause defects in both intra-bead fibres and intra-layers. Indeed, as demonstrated in previous authors’ work [35], there is a technological issue in the CFF process: for each layer printed with carbon fibres (isotropic pattern), the fibres are placed side by side without any additional matrix between the fibre beads. In other words, for a given layer, it can only be either Onyx or fibre; the combination of the two is not possible with the printer setting. For the reasons explained, porosities or voids were probably created in similar locations layer by layer, and these in turn created a preferred way for damage to occur.

This issue is avoided in the other types of samples investigated by changing the orientation of the CF. The elevated temperature of each layer of thermoplastic filament newly deposited on top of the previous layer causes the outer surface to melt again, thereby allowing the new layers to improve matrix-reinforcement interfacial adhesion. Indeed, for both 0/90° and 0/90/45°, the failures are valid and compliant with the standard, considering that they occur in the GM zone. In the former case, the fracture type is lateral, in the latter case it is multi-mode, i.e., it occurs in x, y and z directions.

## 4. Conclusions

In this work, open-hole tensile tests were carried out on composites with Onyx as matrix and carbon fibres as reinforcement, printed by using CFF technology. The influence of isotropic and concentric fibre fill patterns was studied, specifically with three different kinds of hole reinforcement made using the concentric strategy, i.e., no reinforcement, six CF rings and twelve CF rings; and three kinds of stacking sequence made using the isotropic strategy, i.e., [0]_7S_, [0,90,0,90,0,90,0]_S_ and [0,90,+45,−45,0,90,+45]_S_. On the basis of the experimental outcomes, the following conclusions can be drawn:

- The infill strategy adopted to print the carbon fibres strongly influences the mechanical behaviour of open-hole samples. The full Onyx and the hole-reinforced samples show a Lateral Gage Middle failure, while the specimen with CF printed using the isotropic strategy shows several failure modes, depending on the stacking sequence adopted. Open-hole strength and elastic properties are strongly influenced by the strategy. In the isotropic samples, it is possible to reach up to a 633% increment in the open-hole tensile strength and an increment of over 1500% in stiffness.
- The stacking sequence affects the open-hole tensile strength, the stiffness and the failure mode. Concerning the open-hole tensile strength, this decreases as the complexity of the stacking sequence increases. Specifically, the [0,90,0,90,0,90,0]_S_ samples are characterised by a 7% reduction in OHT strength, and the [0,90,+45,−45,0,90,+45]_S_ samples are characterised by a 30% reduction, both in comparison to the [0]_7S_ samples. The stiffness presented the same trend, showing a reduction of 17% in the [0,90,0,90,0,90,0]_S_ samples and a reduction of 33% in the [0,90,+45,−45,0,90,+45]_S_ samples, both in comparison to the [0]_7S_ samples. The results also pointed out a technological issue characterising the process: the printing of unidirectional samples, in the present work [0]_7S_ samples, probably involves defects in the aligned and continuous fibres layer by layer, leading to a longitudinal splitting failure type inside the mechanical grip, which is invalid for open-hole tensile test standards. The [0,90,0,90,0,90,0]_S_ samples are the best case, with an open-hole tensile strength of 220 MPa, a stiffness of 26,000 MPa and a Lateral Gage Middle failure, i.e., the same as the full onyx samples.

These findings can be used to guide the design and manufacturing of 3D-printed composites with carbon fibres for applications that require high strength and durability.

## Figures and Tables

**Table 1:** Experimental campaign.

ID of Samples	Fibre Fill Pattern	Walls to Reinforce	n. of Concentric Fibre Rings	Fibre Angle and Stacking Sequence
Onyx	Concentric	Inner Holes Only	0	-
6C	Concentric	Inner Holes Only	6	-
12C	Concentric	Inner Holes Only	12	-
0°	Isotropic	-	0	[0°]_7S_
0/90°	Isotropic	-	0	[0,90,0,90,0,90,0]_S_
0/90/45°	Isotropic	-	0	[(0/90,+45/−45, 0/90/45)]_S_

## Data Availability

Not applicable.
